# Active management of the third stage of labour in Ethiopia: A systematic review and meta-analysis

**DOI:** 10.1371/journal.pone.0281343

**Published:** 2023-04-20

**Authors:** Gedefaye Nibret Mihretie, Alemu Degu Ayele, Tewachew Muche Liyeh, Fentahun Yenealem Beyene, Bekalu Getnet Kassa, Dawit Tiruneh Arega, Habtamu Gebrehana Belay, Mulugeta Dile Worke

**Affiliations:** 1 Department of Midwifery, College of Medicine and Health Sciences, Debre Tabor University, Debre Tabor, Ethiopia; 2 Department of Midwifery, College of Medicine and Health Sciences, Bahir Dar University, Bahir Dar, Ethiopia; Aga Khan University pakistan, PAKISTAN

## Abstract

**Background:**

Post-partum haemorrhage occurs in over 10% of all births and is the leading cause of maternal mortality, accounting for 25% of all maternal deaths worldwide. Active management of the third stage of labor is the most important intervention for reducing maternal morbidity and mortality by preventing postpartum hemorrhage. Previously, documented primary studies had been great discrepancy, inconsistent results, and there is a lack of comprehensive study. Hence, this systematic review and meta-analysis were intended to assess the prevalence and associated factors of the practice of active management of the third stage of labour among obstetric care providers in Ethiopia.

**Method:**

Cross-sectional studies were systematically searched from January 01, 2010, to December 24, 2020, using PubMed, Google Scholar, HINARI, Cochrane Library, and grey literature. The pooled prevalence of active management of the third stage of labour practice and associated factors was estimated using DerSemonial-Laird Random Effect Model. Stata (version 16.0) was used to analyze the data. The I-squared statistic was used to assess the studies’ heterogeneity. A funnel plot and Egger’s test were used to check for publication bias. A subgroup analysis was performed to minimize the underline heterogeneity depending on the study years and the sample sizes.

**Results:**

Seven hundred fifty articles were extracted. The final ten studies were included in this systematic review, including 2438 participants. The pooled prevalence of practices of active management of the third stage of labour among obstetric care providers in Ethiopia was 39.65% (30.86, 48.45%). Educational status (OR = 6.11, 95%CI, 1.51–10.72), obstetric care training (OR = 3.56, 95% CI: 2.66, 4.45), work experience (OR = 2.17, 95%CI, 0.47, 3.87) and knowledge of active management of the third stage of labour (OR = 4.5, 95% CI: 2.71, 6.28) were significantly associated with active management of the third stage of labour practices.

**Conclusion:**

The practice of active management of the third stage of labour in Ethiopia was low. This study showed that educational status, taking obstetric care training, knowledge of AMTSL, and work experience of obstetric care providers were associated with of practices of active management of the third stage of labour. Therefore, obstetric care professionals should improve their academic level, knowledge, and skills in order to provide useful service to AMTSL and save mothers’ lives. All obstetric care providers should get obstetric care training. Furthermore, the government should increase obstetric care professionals’ educational level.

## Introduction

The third stage of labour is the period directly after the birth of the fetus, which includes the separation and detachment of the placenta from the uterine wall and the complete removal of the placenta and membrane. The third stage of labour is the shortest time, but due to the possibility of profuse hemorrhage, the dangerous and most crucial stage for the birthing woman [1, 2].

Practice of active management of the third stage of labour (AMTSL) is the application of three interrelated but independent components, such as prophylactic administration of a uterotonic drug (oxytocin), controlled cord traction, and uterine massage. Currently, World Health Organization (WHO) recommends active management of the third stage of labour as a critical intervention for postpartum haemorrhage (PPH) prevention, which decreases the occurrence rate of postpartum haemorrhage (PPH) by 60–70% [3].

Every pregnant woman is at risk of PPH because two-thirds of women with PPH have no risk factors [4]. Post-partum hemorrhage (PPH) occurs in over 10% of all births [5] and is the leading cause of maternal mortality, which accounts for 25% of all maternal deaths worldwide [6]. Clinical trials in developed countries show that the use of AMTSL significantly reduces post-partum hemorrhagic disorders [7]. It reduces the relative risk of postpartum hemorrhage by about 60%, compared with physiological (passive) care.

The clinical trial study showed that, routine ’active management is more advantageous than expectant management in terms of reducing blood loss, postpartum hemorrhage, and other serious complications of the third stage of labour [8]. Another systemic review of the global causes of maternal mortality indicated that AMSTL could reduce maternal blood loss by up to 66% [9]. Active management of the third stage of labor is recommended by the International Federation of Gynecologists and Obstetricians (FIGO), the International Confederation of Midwives (ICM), and WHO for all vaginal deliveries [10]. Active management of the third stage of labour reduces the incidence of PPH, the need for blood transfusions, and the use of therapeutic uterotonics [11]. Although the application rates of AMTSL by skilled birth attendants (SBAs) were high, the correct use of AMTSL was only 0.5% to 32% of deliveries [12]. In developing countries, most women who give birth in a health facility do not receive proper care during the third stage of labour [13]. Controlled cord traction reduced the risk of haemorrhage by nearly 50%, and controlled cord traction reduced the risk of hemorrhage by 66% [14].

In Ethiopia, several primary studies have been conducted in various regions to examine the prevalence and associated factors of active management of the third stage of labor among obstetric care providers. These studies had great discrepancies and inconsistent results. Hence, this systematic review and meta-analysis aimed to synthesize the pooled prevalence of the practice of active management of the third stage of labour and its associated factors in Ethiopia. Thus, this study will provide input information for policymakers, maternal care providers, and concerned shareholders for designing prevention strategies, and management of postpartum hemorrhage among women who gave birth at health facilities. Furthermore, the evidence bred from this review can be used as input for researchers who intend to make further investigations in this area. Generally, the findings of this study could help to achieve the sustainable development goal target of reducing maternal and child mortality.

## Methods

### Study settings

This systematic meta-analysis was conducted in Ethiopia. Ethiopia has nine regions: Tigray, Afar, Amhara, Oromia, Somali, Benishangul-Gumuz, Southern Nations Nationalities and People Region (SNNPR), Gambella, Harari, and two administrative states (Addis Ababa City and Dire-Dawa City Administrations).

### Design and protocol of the study

This study includes published and unpublished articles on the prevalence of active management of the third stage of labour practices among obstetric care providers in Ethiopia. The result of this review was developed based on the PRISMA (Preferred Reporting Items for Systematic Review and Meta-Analysis) guidelines [15]. The International Prospective Register of Systematic Reviews has registered the review as a protocol (CRD42021225488).

### Eligibility criteria

#### Study participants

The study participants were obstetric care providers in Ethiopia. They were from all socioeconomic statuses, all ethnic groups, and languages, and were human subjects. This study included all published and unpublished observational studies on active management practices of the third stage of labour and factors affecting practices of active management of the third stage of labor among obstetric care providers in Ethiopia. This review included studies done from January 01, 2010, to December 24, 2020, and was written in English. Studies without full content, anonymous reports, editorials, commentaries, case reports, and qualitative studies were excluded.

#### Types of outcome measures

Practices of active management of the third stage of labor.

### Search strategy and study selection

Both published and unpublished studies conducted about practices of active management of the third stage of labour and its associated factors in Ethiopia were searched. Candidate studies reported in English were identified through an online search of PubMed, HINARI, Google Scholar, and university repositories. Article searches were conducted from November 15, 2020, to December 24, 2020. Different search strategies were used to exhaustively search for studies to be included in this systematic review and meta-analysis. The PECO (Population, Exposure, Comparison, and Outcomes) search format was used in this review to search for pertinent studies. Studies that did not include abstracts and complete text studies were excluded.

#### Population

Obstetric care providers.

#### Exposure

Determinants of active management practices in the third stage of labour (socio-demographics such as age, educational status,) professional-related factors (in-service training, work experience), and knowledge of AMSTL practices.

#### Comparison

The reported reference groups for each determinant factor in each respective study, such as knowledge of active management of the third stage of labour practices among those with a degree or above education versus those who had a diploma, among obstetric care providers.

The studies were exhaustively searched from international databases using comprehensive searching strategies. The following search strategy applied; "Practice"[All Fields] AND “active management” [All Fields] OR "management"[MeSH Terms] AND "management"[All Fields]) AND " third stage labor"[MeSH Terms] OR "labor"[All Fields] AND "stage"[All Fields] OR "third labor stage"[All Fields] OR "stage"[All Fields] AND "labour"[All Fields]) OR "third stage of labour"[All Fields]) AND associated factors [All Fields] OR Determinant [MeSH Terms] OR predictor [All Fields] AND obstetric[All Fields] AND care[All Fields] AND providers[All Fields] AND ("Ethiopia"[MeSH Terms] OR "Ethiopia"[All Fields]). Besides, this, studies were also searched from the reference lists of all included studies to find any other missed studies by our searching strategies. Furthermore, to find relevant unpublished studies, Ethiopian Universities’ digital libraries were searched (S1 File).

### Identification and study selection

All search articles were exported to the Endnote X7 reference manager software and duplicated articles were excluded. The articles were screened by carefully reading the titles and abstracts. Eight authors (GNM, ADA, TML, DTA, FYB, HGB, BGK, and MDA) screened and assessed articles independently. The titles and abstracts of articles clearly stated the outcomes of the review. Then, the full text of the studies was further evaluated based on objectives, methods, participants/population, and critical findings. Any divergences or disagreements were resolved through discussion and consensus based on established criteria.

#### Outcome

Practices of active management of the third stage of labour among obstetric care providers. **The practice of active management of the third stage of labour**; is the application of all three interrelated but independent components immediately after the delivery of the fetus. The components are the administration of a uterotonic drug, controlled cord traction, and uterine massage.

### Quality assessment

The scientific strength and quality of each study were assessed by using the Newcastle-Ottawa Scale quality assessment tool adapted for cross-sectional study quality assessments All authors independently, using the assessment tool, weighted the qualities of each original study. An assessment score that satisfied a 50% quality evaluation criterion was included for analysis (≥5 out of 10). A score difference between the investigators was managed by considering the average score of their quality evaluation outcomes (S2 File).

### Data abstraction

Following the selection of appropriate studies, the authors independently abstracted all necessary data using standardized data extraction form. This form includes the primary author, year of study, study setting, sample size, study design, response rate, prevalence, and specific factors associated with practices of active management of the third stage of labor. For the second objective (factors), the information extraction format was prepared for each specific factor, i.e., obstetric care providers’ training, knowledge of AMSTL, educational status, and work experience. Selected variables had at least two or more studies reporting them as significant factors.

#### The outcome of interest

The practices of active management of the third stage of labor and factors affecting the practices of active management of the third stage of labor were the first and second objectives of the systematic reviews and meta-analysis, respectively.

### Publication bias and heterogeneity

Comprehensive searches (database and manual searches) were used to minimize the risk of bias. The authors’ cooperative work was also critical in reducing bias, selecting articles based on clear objectives and eligibility criteria, deciding the quality of the studies, and extracting and compiling the data. We examine publication bias with a visual inspection of the funnel plot graph qualitatively. Besides, Egger’s correlation tests at a 5% significant level were done to assess the presence of publication bias [16, 17]. Furthermore, to reduce the random variations among the primary studies point estimates and subgroup analysis were done. Heterogeneity across studies and within the studies was evaluated using I-squared statistics with its corresponding p-value.

### Statistical analysis

We used Microsoft Excel for data entry and STATA version 16 software for analysis. The associated factors of practices of active management of the third stage of labour were examined based on eligibility criteria. At least two studies reported at least one associated factor of practices of active management of the third stage of labor in common with their measure of effect. The random-effects model based on the DerSimonian-Laird method was considered to assess for variations between the studies. The results were presented using texts, tables, and forest plots with measures of effect and a 95% confidence interval. Statistical heterogeneity was tested via the I^2^ statistics at a *p*-value of ≤ 0.1 [18].

## Results

### Description of the studies

**Fig 1:**
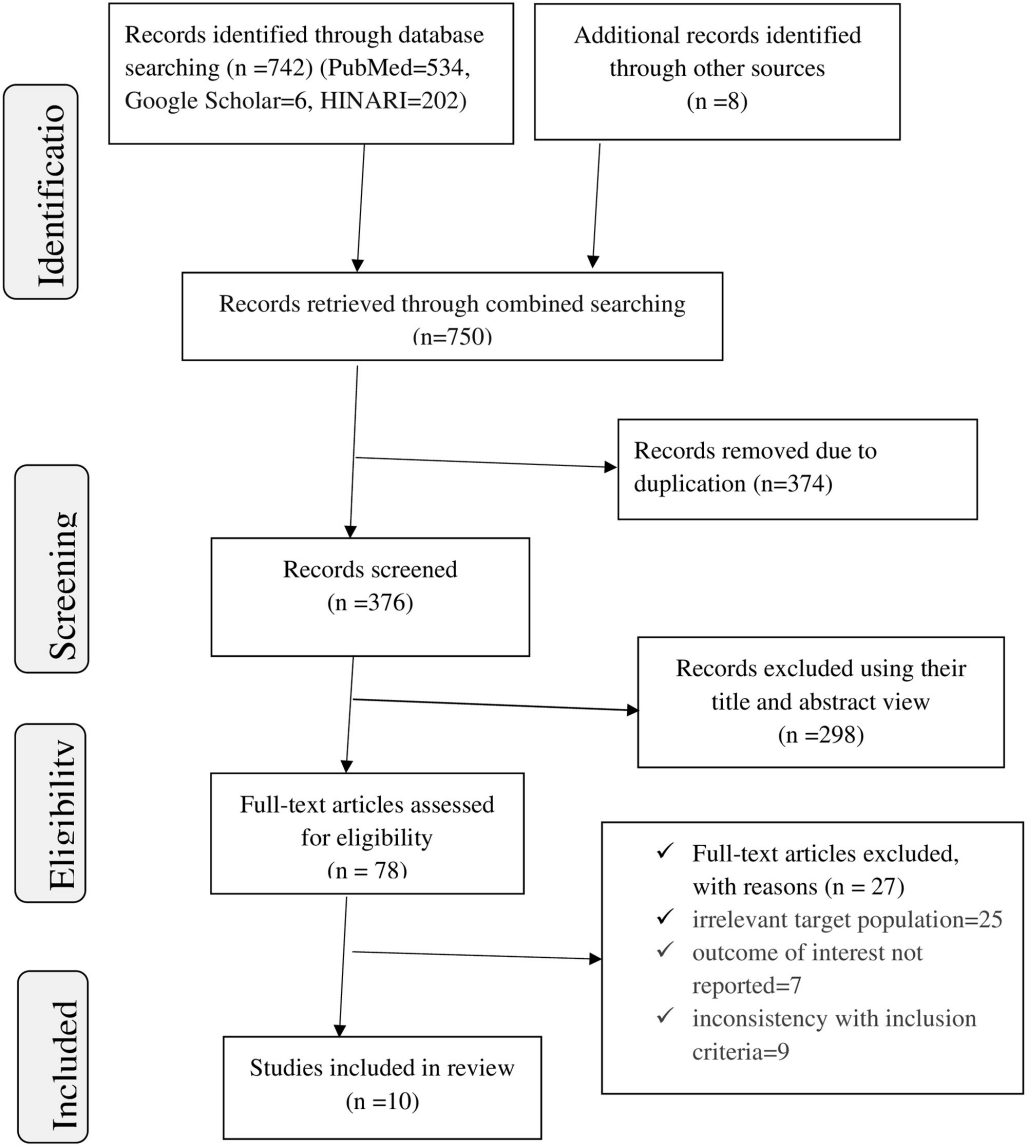
PRISMA flow chart revealing study selection for systematic review and meta-analysis of the practice of active management of the third stage of labor in Ethiopia.

Seven hundred forty-two primary studies were identified by using the major medical and health electronic databases and eight studies from other relevant sources. From 750 identified studies, 374 were excluded after reviewing their titles due to duplication, whereas 368 articles were allowed further screening. Out of these, 298 articles were removed due to irrelevance after being screened based on titles and abstracts. Of the remaining 70 articles, 60 were excluded due to irrelevant target population, inconsistent study report, the outcome of interest not being reported, unavailability of full text, and inconsistency with the predetermined inclusion criteria for the review. Finally, ten studies were used for the systematic review and meta-analysis, with a total population of 2438 (Fig 1).

### Characteristics of the included studies

All ten studies eligible for the systematic review and meta-analysis were cross-sectional study designs and reported in English. The sample size ranges from 76 in Hawassa [19] to 528 in Sidama [20]. Regarding the geographical distribution of studies, four studies were from Southern Nations Nationalities and People Region (SNNPR [19–22], two from Amhara [23, 24], one from Tigray [25], two from Addis Ababa [26, 27], and one from Oromia [28]. Ten of the included studies dealt with practices of active management of the third stage of labor in Ethiopia [19–28] (Table 1).

**Table 1:** Characteristics of included studies reporting the prevalence of practices of active management of the third stage of labor in Ethiopia, 2010 to 2020.

First Author & Study Years	Region	Study setting and Study design	Study population	Parameter studied	Sample size	Response rate	Prevalence (%) with 95% CI
Yaekob R et al. [26]	Addis Ababa	Institutional based Cross-sectional	Midwives	Practices of AMTSL	143	95.1%	51.5(49.32–53.68)
Henok A et al. [27]	Addis Ababa	Institutional based Cross-sectional	Midwives	Practices of AMTSL	143	95.1%	50.7(48.52–52.88)
Lami H et al. [28]	Oromia	Institutional based Cross-sectional	obstetric care provider	Practices of AMTSL	117	100%	30.0(28.00–32.00)
Tenaw Z et al. [19]	SNNPR	Institutional based Cross-sectional	obstetric care provider	Practices of AMTSL	76	95%	16.7(15.07–18.33)
Wake G et al. [25]	Tigray	Institutional based Cross-sectional	Midwives	Practices of AMTSL	285	97.5%	43.5(41.34–45.66)
Adane D et al. [23]	Amhara	Institutional based Cross-sectional	obstetric care provider	Practices of AMTSL	356	100%	61.20(59.07–63.33)
Wudneh A et al. [21]	SNNPR	Institutional based Cross-sectional	obstetric care provider	Practices of AMTSL	180	95%	29.8(27.80–31.80)
Tenaw Z et al. [20]	SNNPR	Institutional based Cross-sectional	obstetric care provider	Practices of AMTSL	528	96.4%	32.8(30.75–34.85)
Bante A et al. [22]	SNNPR	Institutional based Cross-sectional	obstetric care provider	Practices of AMTSL	356	97%	48.1(45.92–50.28)
Molla W et al. [24]	Amhara	Institutional based Cross-sectional	obstetric care provider	Practices of AMTSL	254	91.3%	32.30 (30.26–34.34)

SNNPR = **Southern Nations Nationalities and People Region**

### The practice of active management of the third stage of labour

**Fig 2:**
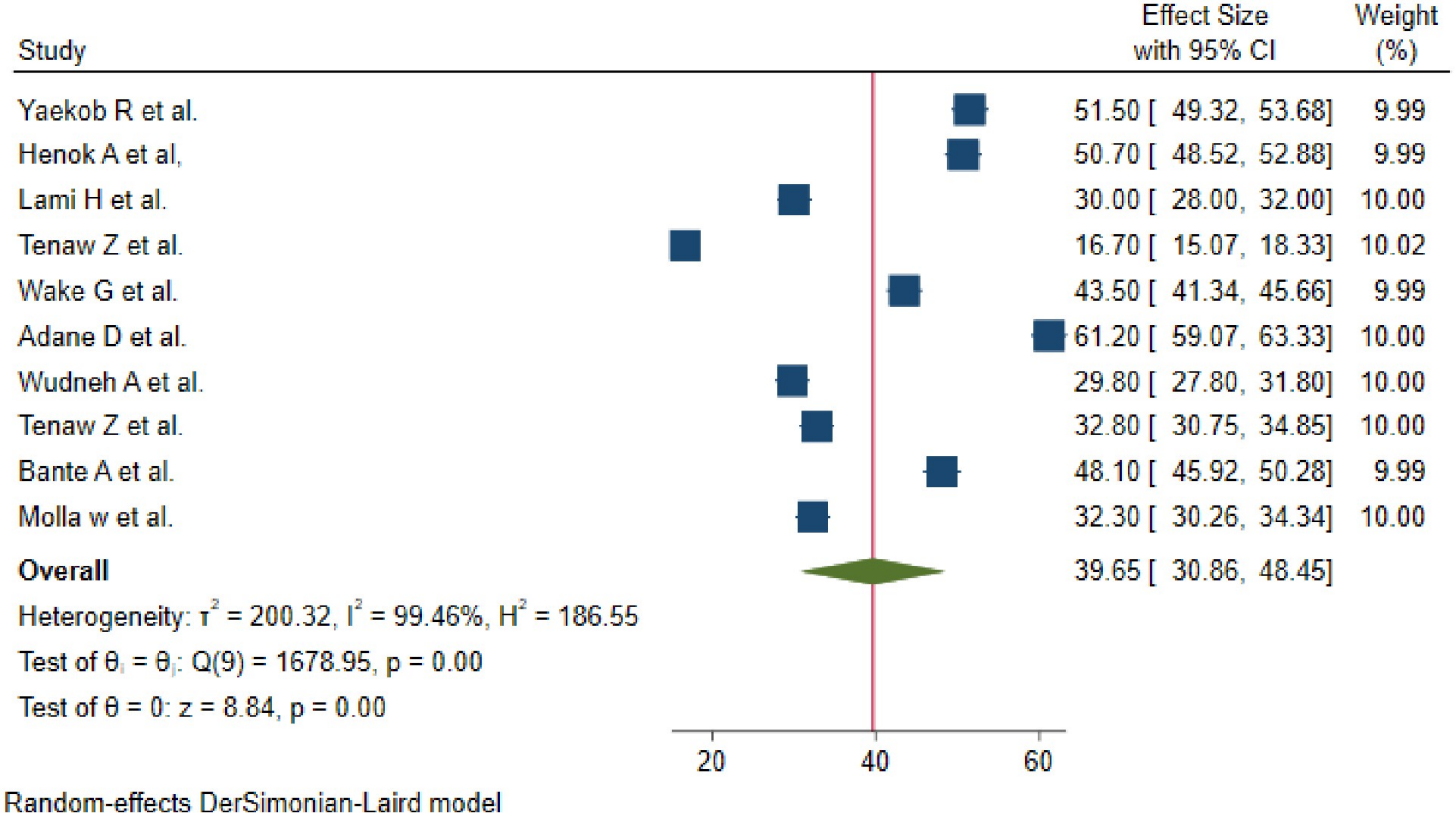
Forest plot of the pooled prevalence of practices of active management of the third stage of labor in Ethiopia.

The overall pooled prevalence of active management practices of the third stage of labour among obstetrics care providers in Ethiopia was 39.65% (95% CI: 30.86–48.45) (Fig 2).

### Heterogeneity and publication bias

All ten studies eligible for meta-analysis dealing with active management practices of the third stage of labour had considerable heterogeneity (I^2^ = 99.46%, *P≤0*.*001*).

**Fig 3:**
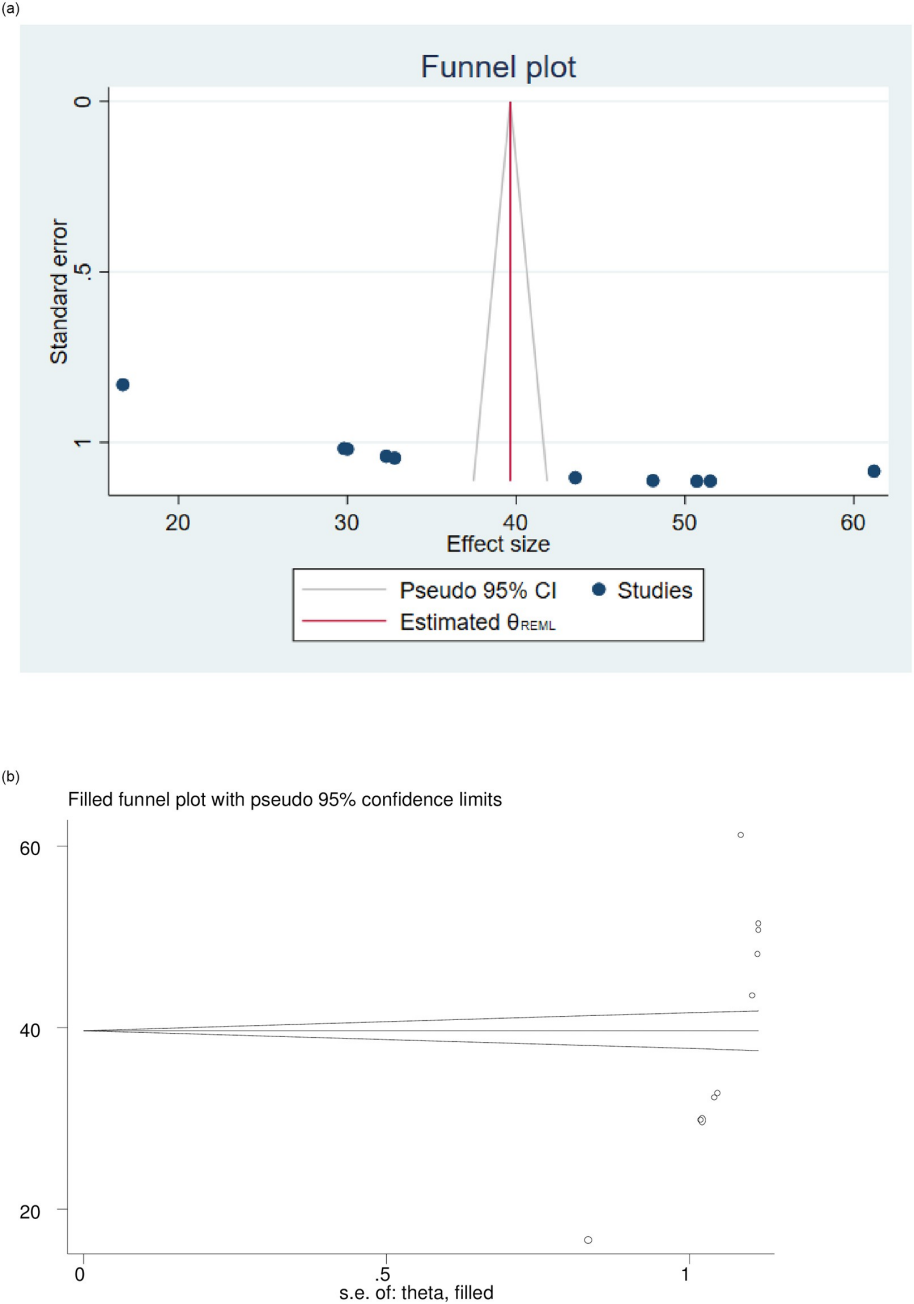
a: Funnel plot for assessing publication bias of the prevalence of practices of active management of the third stage of labor in Ethiopia. b: Funnel plot of the 12 studies’ fill and trim analysis results for adjusting publication bias.

Publication biases among all included studies were examined by using both funnel plots and Egger’s regression test. The results of funnel plots showed an asymmetric shape, which indicates the presence of publication bias (Fig 3a). Egger’s regression test also showed the presence of publication bias across studies (p-value <0.001).

The Duval and Tweedie nonparametric trim and fill analyses were done among the studies dealing with the practice of active management of the third stage of labour. Accordingly, publication bias was corrected when two missed studies were filled in the funnel plot by trimming and filling analysis. After two studies were filled, 12 studies were included and computed via the trim and fill analysis to give the pooled prevalence of 34.85% (95% CI, 25.50–44.21) (Fig 3b).

### Sensitivity analysis

In the current meta-analysis, to determine the potential source of heterogeneity seen in the pooled prevalence of practices of active management of the third stage of labour, the authors did a sensitivity analysis. The sensitivity analysis result indicated that the finding did not rely on a particular study. The pooled prevalence of practices of active management of the third stage of labour varied and ranged from 37.25% (29.11–45.40%) to 42.20% (34.68–49.72%) (Table 2).

**Table 2:** Sensitivity analysis of the pooled prevalence of practices of active management of the third stage of labor.

Study omit	Estimate	95% confidence interval
Yaekob R et al. (2014)	38.33	29.07–47.59
Henok A et al. (2014)	38.42	29.10–47.74
Lami H et al. (2019)	40.72	31.05–50.39
Tenaw Z et al. (2015)	42.20	34.68–49.72
Wake G et al. (2018)	39.22	29.53–48.91
Adane D et al. (2018)	37.25	29.11–45.40
Wudneh A et al. (2018)	40.74	31.08–50.40
Tenaw Z et al. (2015)	40.41	30.65–50.17
Bante A et al. (2018)	38.71	29.23-48-29
Molla W et al. (2018)	40.46	30.72–50.21

### Subgroup analysis

**Fig 4:**
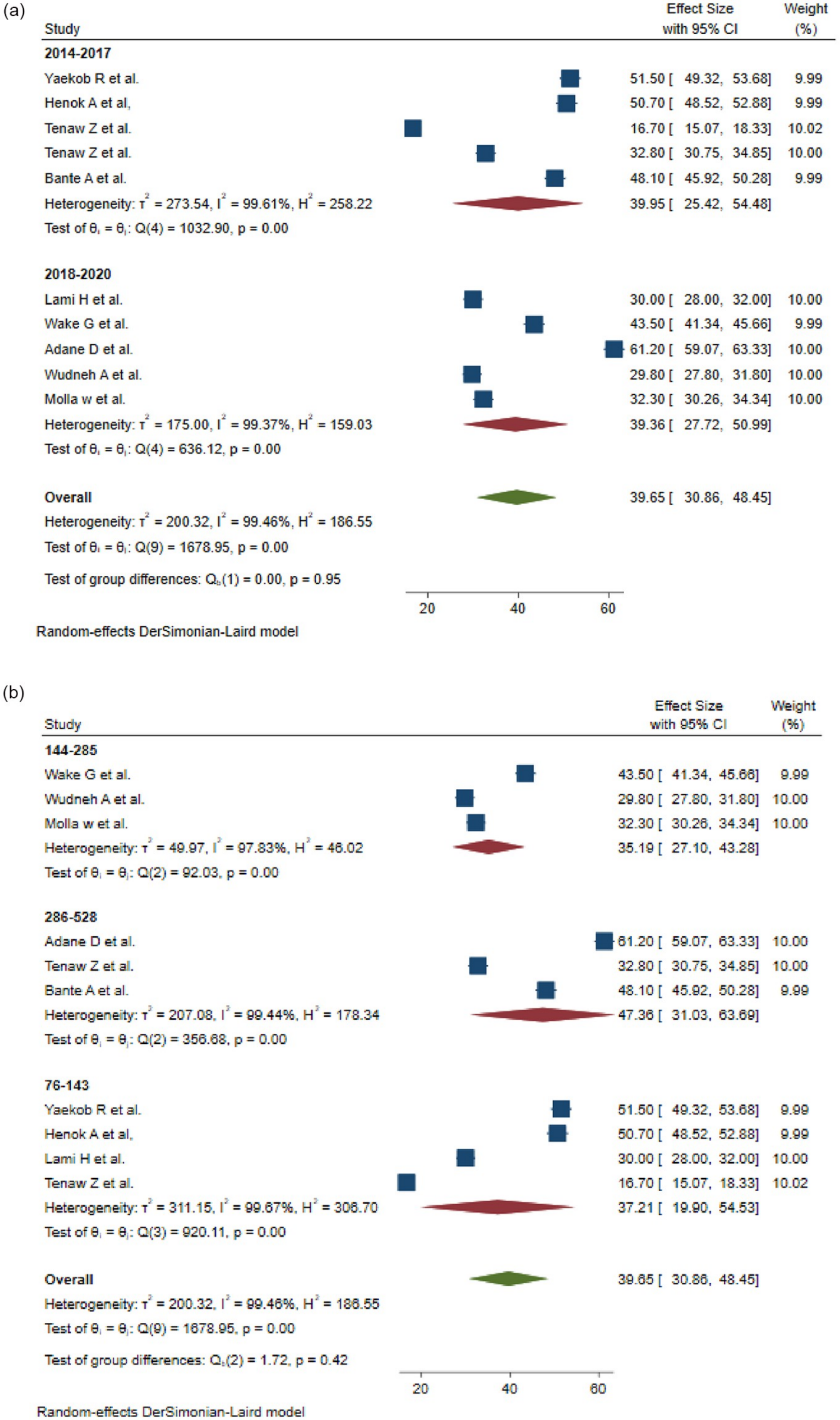
a: Subgroup analysis of the pooled prevalence of practices of active management of the third stage of labor based on the study period. b: Subgroup analysis of the pooled practices of the prevalence of active management of the third stage of labor based on the sample size.

A subgroup analysis based on study years and sample sizes was done. Based on study years 2010–2017 years and 2018–2020 years, the pooled prevalence was 37.92% (95%CI, 20.62–55.21) and 40.81% (95%CI, 30.79–50.84), respectively (Fig 4a). Subgroup analysis using sample size, sample sizes between 286 and 528 sample sizes have a higher pooled prevalence of 47.36 (95% CI, 31.01%–63.69) (Fig 4b).

### Factors associated with the practice of active management of the third-stage labour

Educational status, training, knowledge of AMTSL practices, and professional work experience were significantly associated with practices of active management of the third stage of labour.

### The association between training and practice of active management of the third stage of labour

**Fig 5:**
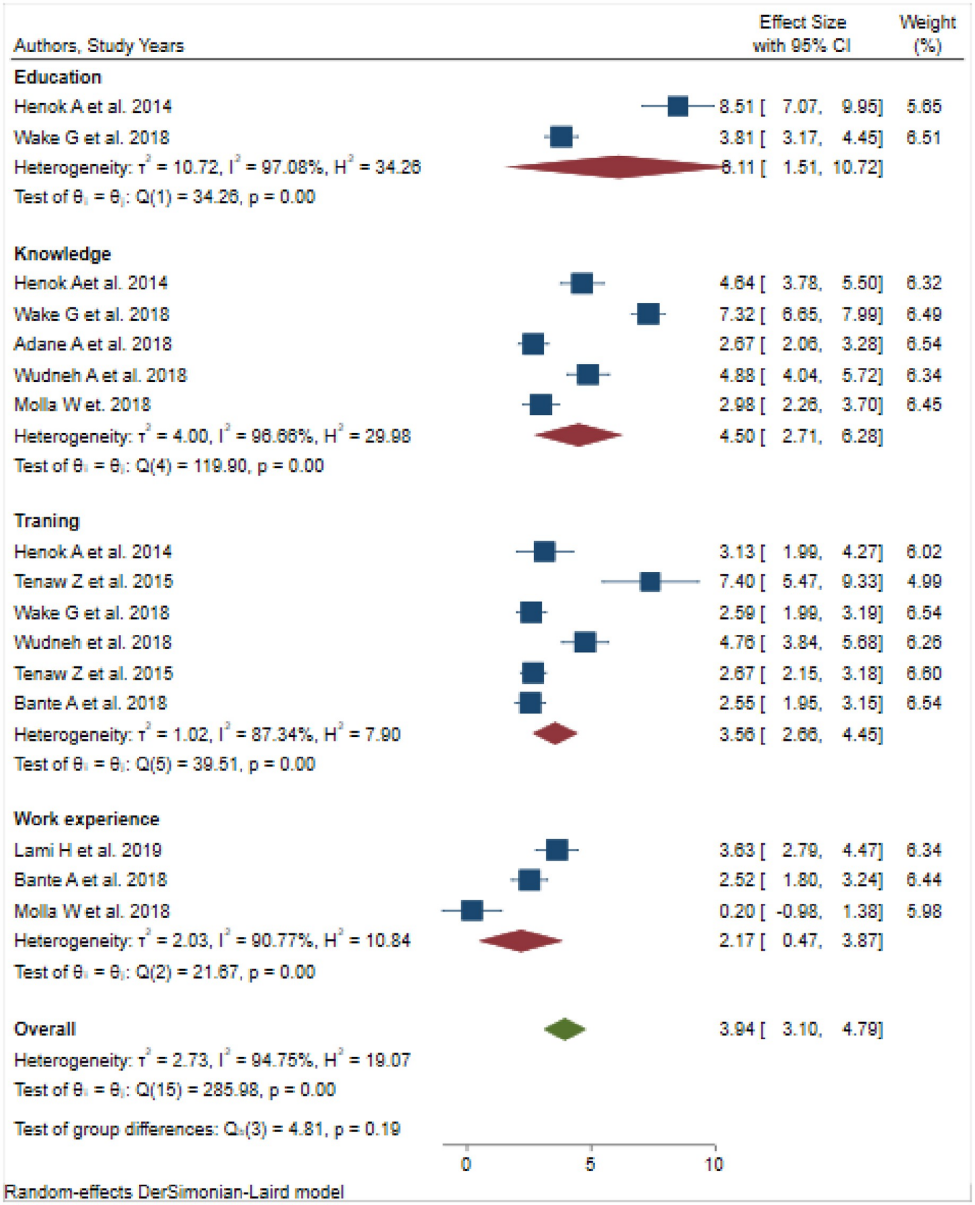
Forest plot of the association between training, educational status, knowledge of active management practices of the third stage of labour, and work experience with the practice of active management of the third stage of labour in Ethiopia.

Six primary articles revealed that training was significantly associated with practices of AMTSL [19–22, 25, 27]. Obstetric care providers who took training were three times (OR = 3.56, 95%CI, 2.66–4.45) more likely to practice on AMTSL than obstetric care providers who did not take training. The heterogeneity test indicated *I*^*2*^ *= 87*.*34%*, *P = 0*.*00)*, hence the random-effect model was applied for analysis (Fig 5).

### The association between educational status and the practice of active management of the third stage of labour

Two primary studies reported that having a degree and above educational status was strongly associated with AMTSL practices [25, 27]. When compared with those obstetric care providers who had a degree and above education, six times (OR = 6.11, 95%CI, 1.51–10.72) were more likely to practice AMTSL. The heterogeneity test showed that *I*^*2*^ *= 97*.*08%*, *P = 0*.*00*, hence a random-effect model was used for analysis (Fig 5).

### The association between knowledge and practice of active management of the third stage of labor

Five primary studies showed that knowledge about AMTSL was a significant predictor of practices of AMTSL [21, 23–25, 27]. Obstetric care providers who knew AMSTL practices four times (OR = 4.50, 95%CI, 2.71–6.28) more liked to practice than their counterparts. The heterogeneity test showed an I^2^ value of 96.66%, *P = 0*.*00*, and we used the random-effect model for analysis (Fig 5).

### The association between work experience and practice of active management of the third stage of labour

Three primary articles identified that work experiences were significantly associated with the practices of AMTSL [22, 24, 28]. Obstetric care providers who had two years or more of clinical obstetric care experience in the obstetric department were 2 times (OR = 2.17, 95%CI, 0.47–3.87) more likely to practice on AMTSL as compared to obstetric care providers who had less than two years of work experience. The heterogeneity test indicated I^2^ = 90.77%, *P = 0*.*00* hence the random-effect model was applied for analysis (Fig 5).

## Discussion

Practicing active management of the third stage of labour has been successful in reducing post-partum haemorrhage [29]. The FIGO guideline strongly recommends that every obstetrical care provider at birth needs to have knowledge, skills, and critical judgment to carry out active management of the third stage of labour appropriately [30]. This systematic review and meta-analysis aimed to assess the pooling prevalence of active management practice of the third stage of labour and its determinant factors in Ethiopia. The pooled prevalence of the practice of active management of the third stage of labour in Ethiopia was 39.65% (30.86–48.45%). This finding was higher than the study conducted in Rwanda (15.9%) and Tanzania (7.0%) [31, 32], respectively. This finding was also in line with the study conducted in Nigeria, 41.0% [33]. The difference might be attributed to variations in the study period, sample size, and the socio-cultural condition of the society.

Obstetric care providers who did take training about obstetric care were 3 times more likely to practice active management of the third stage of labour compared with those who did not take training. Including and ensuring successful pre-service and in-service training of active management of the third stage of labour will increase obstetric care providers’ knowledge and skills for delivery of the services [30]. There was other evidence that indicated training health professionals improve knowledge, skills, attitudes, and increases care processes [34]. The educational status of obstetric care providers was found to be a predictor of the practice of AMTSL. This study showed that obstetric care providers with a bachelor’s degree or higher were more likely to engage in the practice of active management of the third stage of labor than obstetric care providers who had a diploma.

The odds of practicing active management of the third stage of labour (AMTSL) were higher among participants who had good knowledge of active management of the third stage of labour compared with participants who had poor knowledge of AMTSL. The World Health Organization directs that maternal care providers should obtain refreshment training or upgrades every three to five years. Knowing active management of the third stage of labour improves the practice of AMSTL.

Two or more work experience of obstetric care providers were significantly associated with active management of the third stage of labour than obstetric care providers who had less than two years of work experience. As we know, various types of medical errors have occurred, especially in resource-limited countries, because of poor knowledge and lack of experience-sharing practices among health professionals. Work experiences improve healthcare workers’ knowledge and provide evidence-based healthcare services through their routine activities over time.

## Conclusion

The prevalence of active management of the third stage of labour practice was low. The education status of obstetric care providers, obstetric care training, knowledge of AMTSL, and work experience were predictors of practices of active management of the third stage of labour. Therefore, obstetric care professionals should improve their academic level, knowledge, and skills in order to provide useful service to AMTSL and save mothers’ lives. All obstetric care providers should get obstetric care training. Furthermore, the government should increase obstetric care professionals’ educational level.

## Limitations of the study

Although the study was intended to assess the magnitude of active management of the third stage of labour in Ethiopia, we could not get studies from all regions of the country, which might affect its representativeness. Due to the country’s shortage of studies, small numbers of studies were used to analyze some variables’ pooled effects. However, we have used both published and unpublished studies, which might reduce the risk of publication bias.

## Supporting information

S1 File A searching strategy for active management of the third stage labour practices and associated factors among obstetric care providers in Ethiopia, 2020/21. (DOCX)

S2 File Newcastle-Ottawa quality assessment scale for cross-sectional studies to assess for active management of third stage labour practices among obstetric care providers in Ethiopia, 2020/21. (DOCX)

S1 Checklist PRISMA 2009 checklist. (DOC)

## Abbreviations

AMTSL: Active Management of the Third Stage of Labour
FIGO: International Federation of Gynecologists and Obstetricians
ICM: International Confederation of Midwives
PPH: Post-partum Hemorrhage
WHO: World Health Organization
