# Supplementary material for: Active management of the third stage of labour in Ethiopia: A systematic review and meta-analysis
Source: PLoS One. 2023 Apr 20;18(4):e0281343. doi: 10.1371/journal.pone.0281343 (PMC10118110; doi:10.1371/journal.pone.0281343)
Supplement: S1 File — (DOCX) [file pone.0281343.s001.docx]

Supplementary file **1**: A searching strategy for active management of the third stage labour practices and associated factors among obstetric care providers in Ethiopia, 2020/21.

| Databases | Searching terms | Number of studies |
| --- | --- | --- |
| PubMed | "Practice"[All Fields] AND “active management” [All Fields] OR "management"[MeSH Terms] AND "management"[All Fields]) AND "third stage labor"[MeSH Terms] OR "labor"[All Fields] AND "stage"[All Fields] OR "third labor stage"[All Fields] OR "stage"[All Fields] AND "labour"[All Fields]) OR "third stage of labour"[All Fields]) AND associated factors [All Fields] OR Determinant [MeSH Terms] OR predictor [All Fields] AND obstetric[All Fields] AND care[All Fields] AND providers[All Fields] AND ("Ethiopia"[MeSH Terms] OR "Ethiopia"[All Fields]). | 534 |
| Google Scholar | “Active management” AND “third stage” AND “labour” AND “associated” AND “factors” AND “among” AND “Care giver” AND “Ethiopia” | 6 |
| HINARI | Practice and factors associated with active management of the third stage of labour among obstetric care providers in Ethiopia | 202 |
| Others databases |  | 8 |
| Total retrieved |  | 757 |
| Included |  | 10 |
