# Supplementary material for: Active management of the third stage of labour in Ethiopia: A systematic review and meta-analysis
Source: PLoS One. 2023 Apr 20;18(4):e0281343. doi: 10.1371/journal.pone.0281343 (PMC10118110; doi:10.1371/journal.pone.0281343)
Supplement: S2 File — (DOCX) [file pone.0281343.s002.docx]

Supplementary file 2: Newcastle-Ottawa Quality Assessment Scale for cross-sectional studies to assess for active management of third stage labour practices among obstetric care providers in Ethiopia, 2020/21.

| Authors | Representatives | Sample size | None-responders | Ascertainment | Comparability | Outcome | Quality score |
| --- | --- | --- | --- | --- | --- | --- | --- |
| Yaekob R et al. (2014) | 1 | 1 | 1 | 1 | 1 | 1 | 6 |
| Henok A et al. (2014) | 1 | 1 | 1 | 2 | 1 | 1 | 7 |
| Lami H et al. (2019) | 2 | 1 | 1 | 2 | 1 | 1 | 8 |
| Tenaw Z et al. (2015) | 1 | 1 | 1 | 1 | 1 | 1 | 7 |
| Wake G et al. (2018) | 2 | 1 | 1 | 1 | 1 | 1 | 7 |
| Adane D et al. (2018) | 1 | 2 | 1 | 1 | 2 | 1 | 8 |
| Wudneh A et al. (2018) | 1 | 1 | 1 | 2 | 1 | 1 | 8 |
| Tenaw Z et al. (2015) | 1 | 2 | 1 | 1 | 2 | 1 | 8 |
| Bante A et al. (2018) | 2 | 2 | 1 | 1 | 1 | 1 | 8 |
| Molla W et al.(2018) | 1 | 2 | 1 | 2 | 1 | 1 | 8 |

Interpretation of the score

Very Good Studies: 9-10 points

Good Studies: 7-8 points

Satisfactory Studies: 5-6 points

Unsatisfactory Studies: 0 to 4 point
